# Removal of Metal Nanoparticles Colloidal Solutions by Water Plants

**DOI:** 10.1186/s11671-016-1742-9

**Published:** 2016-11-25

**Authors:** Olga Olkhovych, Nataliia Svietlova, Yevheniia Konotop, Olena Karaushu, Svitlana Hrechishkina

**Affiliations:** Educational and Scientific Centre “Institute of Biology and Medicine”, Taras Shevchenko National University of Kyiv, 64/13, Volodymyrska Street, Kyiv, 01601 Ukraine

**Keywords:** Aquatic plants, Water macrophytes, Metal nanoparticles, Phytoremediation

## Abstract

The ability of seven species of aquatic plants (*Elodea canadensis*, *Najas guadelupensis*, *Vallisneria spiralis* L., *Riccia fluitans* L., *Limnobium laevigatum*, *Pistia stratiotes* L., and *Salvinia natans* L.) to absorb metal nanoparticles from colloidal solutions was studied. It was established that investigated aquatic plants have a high capacity for removal of metal nanoparticles from aqueous solution (30–100%) which indicates their high phytoremediation potential. Analysis of the water samples content for elements including the mixture of colloidal solutions of metal nanoparticles (Mn, Cu, Zn, Ag + Ag_2_O) before and after exposure to plants showed no significant differences when using submerged or free-floating hydrophytes so-called pleuston. However, it was found that the presence of submerged hydrophytes in aqueous medium (*E. canadensis*, *N. guadelupensis*, *V. spiralis* L., and *R. fluitans* L.) and significant changes in the content of photosynthetic pigments, unlike free-floating hydrophytes (*L. laevigatum*, *P. stratiotes* L., *S. natans* L.), had occur. Pleuston possesses higher potential for phytoremediation of contaminated water basins polluted by metal nanoparticles. In terms of removal of nanoparticles among studied free-floating hydrophytes, *P. stratiotes* L. and *S. natans* L. deserve on special attention.

## Background

Rapid involvement of nanotechnology products in different areas of production [1–3] results in their widespread with the industrial emissions into the environment, especially water. As the environmental risks of metal nanoparticles use today is not exactly clarified [4, 5], the possible methods of their removal from the water environment, in order to maintain quality of natural water resources and biodiversity of aquatic ecosystems, must be found out in advance.

It is known that aquatic plants can rapidly absorb water with different pollutants [6–8], so they are widely used in phytoremediation [9–11]. Phytoremediation refers to the technologies that use living plants to remove hazardous chemicals from water, air, and soil. The advantages of phytoremediation are a high remediation level that is not compromised to the physical and chemical methods, low cost, environmental safety, the possibility of further extraction of contaminants from the green mass of plants [12, 13], and monitoring the cleaning process. There is a positive experience and technical methods of phytoremediation of natural reservoirs using aquatic macrophytes for water purification in world practices [14–16], which, unfortunately are restrained in Ukraine due to the lack of relevant solutions and recommendations related to the accumulative capacity of plants and their physiology under pollution.

Thus, the search for species of aquatic plants that would be resistant to the pollution and at the same time capable of removing metal nanoparticles is an important task for phytoremediation of technical basins. Therefore, the aim of our study was screening of different types of aquatic plants for their ability to extract metals from water in order to design new types of water treatment facilities using aquatic plants resistant to contamination.

## Methods

Seven species of aquatic plants, including three types of free-floating on the water surface hydrophytes, so-called pleuston—*Limnobium laevigatum* (Humb. & Bonpl. Ex Willd.), *Pistia stratiotes* L., and *Salvinia natans* (L.) All. and four species of submerged hydrophytes—*Elodea canadensis* Michx., *Najas guadelupensis* (Spreng.) *Magnus*, *Vallisneria spiralis* L., and *Riccia fluitans* L. were used in our study.

All plants were grown in lab pools in Educational and Scientific Centre “Institute of Biology and Medicine” in aquariums (40–60 L) in tap water under optimal conditions: lighting—6000 lx, light period—12 h, water temperature 19–25 °C, and water pH 7.8.

Colloidal solutions of nanoparticles of metals used in the research were developed by the Department of Technology of Materials and Structural Materials of National University of Life and Environmental Sciences of Ukraine by dispersing granules of manganese, copper, zinc, and silver by pulses of electric current with an amplitude of 100–2000 A in the water [17]. The maximum size of nanoparticles does not exceed 100 nm.

**Fig. 1:**
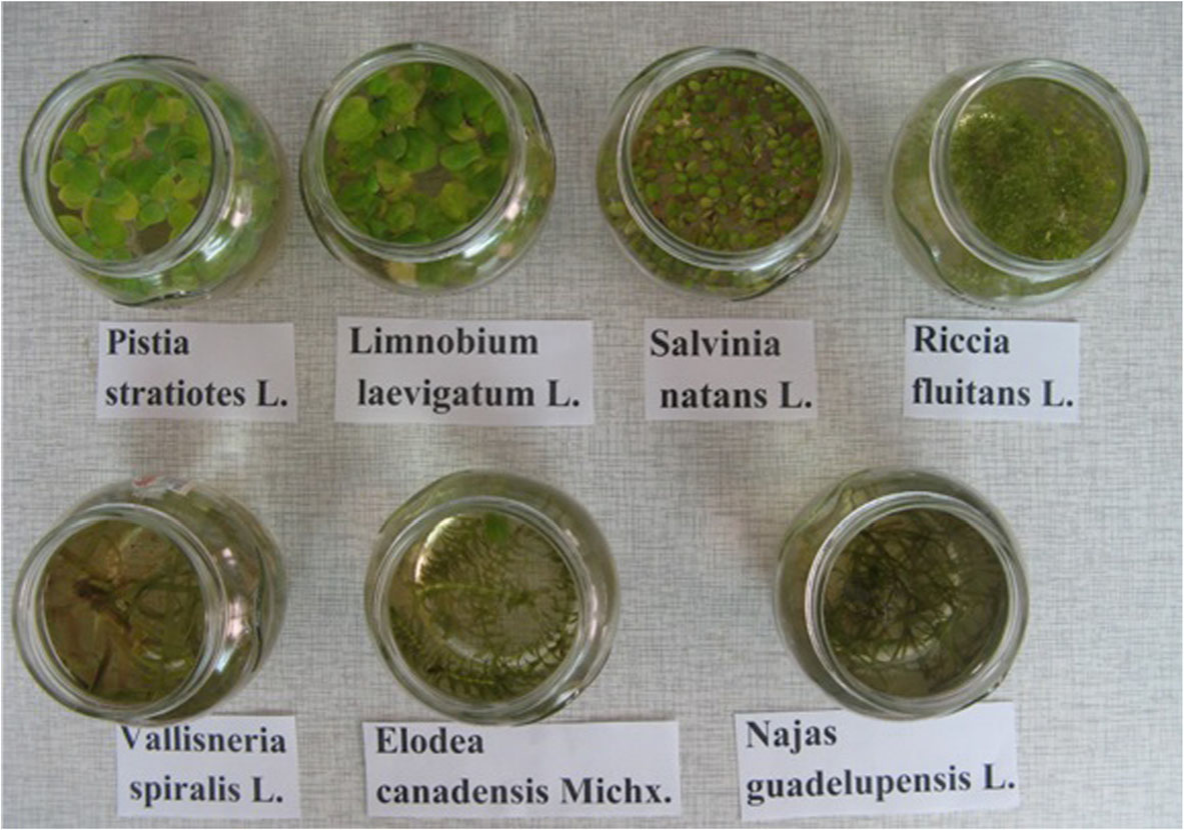
The appearance of aquatic plants exposed to the mixtures of colloidal solutions of metal nanoparticles on the 7th day

The plants were exposed to the solution of settled tap water (at a rate of 1 g per 100 mL of water) with the mixture of colloidal solutions of metal nanoparticles (Mn—0.75 mg L^−1^, Cu—0.37 mg L^−1^, Zn—0.44 mg L^−1^, Ag + Ag_2_O—0.75 mg L^−1^) for 7 days (Fig. 1).

The visual inspection of plants was performed on the 7th day, followed by the determination of plant pigments and content of the studied elements in the water used for plants growth.

Determination of pigments was carried out by standard methods. Pigments were extracted using 96% ethanol. The extracts were analyzed on “Shimadzu UV-1800” spectrophotometer at wavelengths of 440, 644, and 662 nm. The content of pigments was calculated per 1 g dry matter. The content of metal nanoparticles in water samples was determined by ICP-spectrometry using ICAP 6300 Duo MEC (USA) emission spectrophotometer.

Statistical data analysis was conducted using Microsoft Office Excel software. Results were considered significant (by Student’s *t* test) at significance level *p* ≤ 0.05. The number of biological and analytical replications in the experiment is at least threefold.

## Results and Discussion

**Fig. 2:**
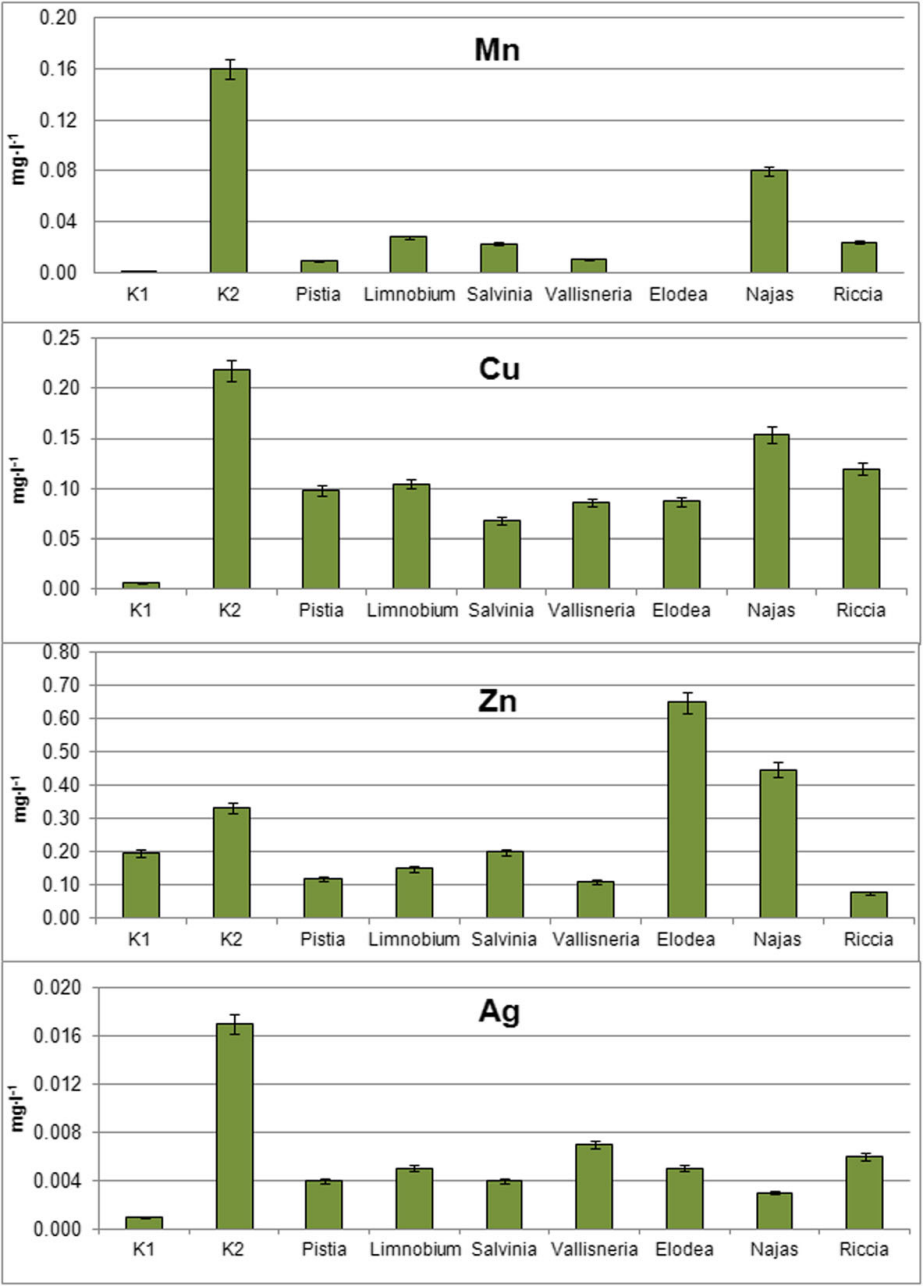
The content of elements in the water after exposure of hydrophytes after 7 days (K1—settled tap water, K2—settled tap water with the addition of colloidal solutions of metal nanoparticles)

In order to ensure that the selected plants are suitable for phytoremediation—and can clean contaminated water environment, it was necessary to find out whether the studied hydrophyte species are capable to remove metal nanoparticles from water, for what the series of experiments in which studied species were submerged into water with the added mixture of colloidal solutions of nanoparticles of metals were conducted. After the 7 days exposure, the plants were removed from the solutions, which were subsequently analyzed for the remaining content of the studied metals (Fig. 2).

It was found that all the studied species were able to absorb metal nanoparticles and remove them from aqueous solutions. The differences in extraction of individual metal nanoparticles from aqueous solutions by different types of hydrophytes as well as plant species specificity regarding the studied metal nanoparticles were revealed.

*E. canadensis* (100%), *P.stratiotes* (94%), and *V. spiralis* (93%) had shown the highest ability to remove manganese nanoparticles from mixtures of colloidal solutions of investigated four nanoparticles of metals. The high capacity for Mn nanoparticles removal from aqueous solution also had *S. natans* (86%), *R. fluitans* (85%), and *L. laevigatum* (82%). The worst Mn nanoparticles extraction was observed in variant with *N. guadelupensis* (50%).

Removal of copper nanoparticles by studied water plants had some specific features. The highest removal rates were observed in the variants with *S. natans* (69%), *V. spiralis* (61%), and *E. canadensis* (60%), and slightly lower in the presence of *P. stratiotes* (55%) and *L. laevigatum* (52%), while other species had removed less than half of this element from the solution with the worst results obtained in the variant with *N. guadelupensis* (30%).

Significant extraction of zinc nanoparticles from mixture of investigated colloidal solutions of nanoparticles was observed in five variants. In this respect, the best Zn accumulators were *R. fluitans* (77%), *V. spiralis* (67%), and *P. stratiotes* (65%). Lower ability to extract was observed in variants with *L. laevigatum* (56%) and *S. natans* (40%). Unexpected results were obtained for *E. canadensis* and *N. guadelupensis*, where the Zn content in water had increased, relatively to the initial reference value (in *N. guadelupensis* 34% and *E. canadensis* up to 95%). This unexpected result can be explained by the destruction of enzyme systems including Zn (carbonic anhydrase), which led to its exit outside the cells into the aqueous solution the mechanism of which needs further research. No other studied metal nanoparticles had shown their content increase in aqueous solution with respect to the initial reference value.

Quantitative argentum content was minimal after exposure of *N.guadelupensis* on colloidal solutions of nanoparticles (metal content had decreased by 82%). Significant results were also obtained for Ag uptake by such plants as *P. stratiotes* and *S. natans* (76%) and *L. laevigatum* and *E. canadensis* (71%). Slightly lower values were observed in variants with *R. fluitans* (65%) and *V. spiralis* (59%). Nevertheless, all these plants can be attributed as Ag accumulators and can be used for phytoremediation.

The results obtained have revealed the lack of significant differences between submerged and free-floating hydrophytes in terms of removal of metal nanoparticles from aqueous solution. Different plants possess the specific ability to extract certain elements. Among all studied metal nanoparticles, *P. stratiotes* and *S. natans* have most advantages on metal removal. Not far less potential to extract nanoparticles from aqueous solutions has *L. laevigatum*. Considering the specificity of aquatic plants species to the studied individual metal nanoparticles, the h performance in respect of Mn removal among submerged hydrophytes had showed *E. canadensis*, respectively, for Cu—*V. spiralis* and *E. canadensis*, Zn—*R. fluitans*, and Ag—*N. guadelupensis*, while among free-floating hydrophytes, the highest removal rates for Mn were noticed in variants with *P. stratiotes*, Cu—*S. natans*, Zn—*P. stratiotes*, and Ag—*P. stratiotes* and *S. natans. N. guadelupensis* showed the worst performance according to the accumulation of three elements, Mn, Cu, and Zn—50, 30, and 34%, respectively. However, *N. guadelupensis* had the best results in Ag removal—82%, thus indicating the possible use of this species for phytoremediation. Probably, further research with the establishment of threshold concentrations for the survival of *N. guadelupensis* will give more precise results.

Our data, concerning extraction of nanoparticles of metals (Mn, Cu, Zn, Ag) by free-floating hydrophytes, is well consistent with these of other scientists declared for the ability to remove Mn, Cu, Zn, and Ag in ionic form, especially by *Pistia* [18–21] and *Salvinia* [22, 23]. There is also an evidence that increasing concentrations of metals above the threshold may result in damage to plants used for phytoremediation, which led to necrotic changes; chlorosis; damage of chloroplasts, stomata cells, and trichomes; and reducing biomass, content of chlorophyll, protein, free amino acids, RNA, and DNA which should be considered when creating water treatment facilities. That is why establishment of plant resistance to the specific metals and their threshold concentrations is receiving much attention nowadays [24–27].

*Pistia* belongs to the alien plant species, not typical for water basins of Ukraine, besides it is even claimed to be the dangerous invasive species [28], so it can be recommended for phytoremediation only in closed technical water pools. However, another species, *Salvinia*, belongs to the indigenous, although quite rare (listed in the Red Book of Ukraine) species, so it can be used even in open natural water basins.

Physiological state of the plant, as well as the content of photosynthetic pigments, is an important marker, used for characterization of the overall state of plant’s metabolism. These parameters have been studied to determine the stability of aquatic plants in the presence of metal nanoparticles colloidal solutions in water. On the 7th day of experiment, the visual examination of plants was carried out and differences of all four species of submerged hydrophytes (*E. canadensis, N. guadelupensis, V. spiralis,* and *R. fluitans*) relatively to control samples were recorded. First of all, this applies to the color and water status of plant, its turgidity. The colors of leaves and stems have been darker, plants had lost their normal shape, turgor pressure in tissues was low, leaves became soft and were hanging from the stems. In free-floating hydrophytes (*L. laevigatum, P. stratiotes*, *and S. natans*), only partial leaves yellowing was observed, without any marked changes in the form of plants or softening of their vegetative organs (Fig. 1).

**Fig. 3:**
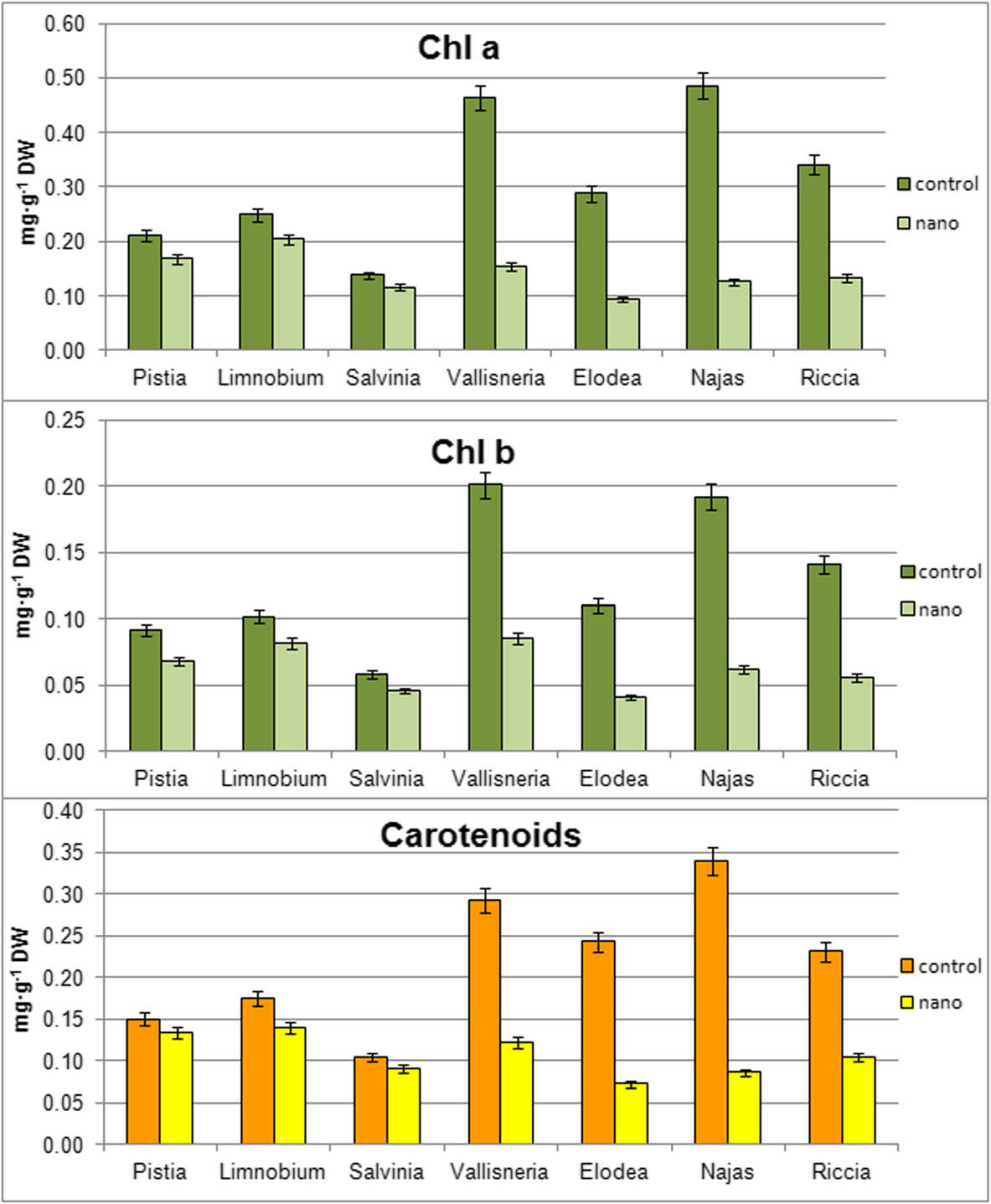
The content of pigments in aquatic plants under the influence of a mixture of colloidal solutions of metal nanoparticles on the 7th day

To determine the causes of color changes that are associated with vitality and productivity of plants, the content of photosynthetic pigments in all investigated plants was determined. The research results are presented in Fig. 3.

Numerous reports deal with the stimulating effect of metal nanoparticles on the chlorophyll content in vascular plants [29–31]. Results of our study had showed that metal nanoparticles negatively affected the content of pigments (chlorophylls and carotenoids) in all studied plants, but the effect was much stronger in submerged hydrophytes than in free-floating plants. The contents of all pigments compared to the controls had decreased. Thus, the content of chlorophyll *a* in *N. guadelupensis* decreased nearly in 4 times (from 0.485 to 0.126 mg g^−1^), while in *E.canadensis* and *V. spiralis*, the numbers were lower in ~3 times (from 0.288 to 0.093 mg g^−1^ and from 0.463 to 0.153 mg g^−1^, respectively) and *R. fluitans*—2.6 times (from 0.342 to 0.133 mg g^−1^). Content of chlorophyll *a* in free-floating hydrophytes had decreased only by 15-20% (in *L. laevigatum* from 0.249 to 0.204 mg g^−1^, in *P. stratiotes* from 0.212 to 0.168 mg g^−1^, respectively and in *S. natans* from 0.138 to 0.116 mg g^−1^).

The content of chlorophyll *b* in submerged hydrophytes had declined less than the content of chlorophyll *a*, namely in *N. guadelupensis*—in more than 3 times (from 0.192 to 0.062 mg g^−1^), 2.7 times in *E. canadensis* (from 0.110 to 0.041 mg g^−1^), 2.5 times in *R. fluitans* (from 0.141 to 0.056 mg g^−1^), and 2.4 times in *V. spiralis* (from 0.201 to 0.085 mg g^−1^). In free-floating plants, the content of chlorophyll *b* had decreased only by 20–27% (in *L. laevigatum* from 0.102 to 0.082 mg g^−1^, in *P. stratiotes* from 0.092 to 0.068 mg g^−1^, and in *S. natans* from 0.058 to 0.046 mg g^−1^).

**Fig. 4:**
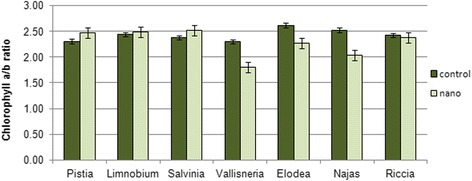
The chlorophyll *a/b* ratio in aquatic plants under the influence of a mixture of colloidal solutions of metals nanoparticles on the 7th day

It is known that chlorophyll *a*/chlorophyll *b* ratio can be used as indirect indicator of the quantity of the light-harvesting complex II of photosystem II [32]. Our data reports that this index was lower in submerged hydrophytes (*V. spiralis*, *E. canadensis*, *N. guadelupensis*) epoxed to the treatment with metal nanoparticles, mainly on the background of a general reduction of chlorophyll content due to the decrease of chlorophyll *a* content (Fig. 4). Chlorophyll *a*/chlorophyll *b* ratio was slightly higher in pleuston (*P. stratiotes*, *S. natans*), that, combining with the lower reduction rates of chlorophylls is an indicator of possible less damage of photosystem II.

Usually, the reduction of chlorophyll *a* content in plants with minor damage causes an increase in the content of carotenoids. But in our study, the effect of metal nanoparticles had led to the decline of carotenoids content as well, and this decrease had matched the decline of chlorophyll content—stronger effect was observed in submerged hydrophytes than in pleuston.

Carotenoids perform many important functions in plant organism. They are involved in photosynthesis, growth, morphogenesis, and reproduction. Serving as supporting pigments of photosynthetic apparatus, carotenoids absorb photons in two peaks—blue-violet and blue spectral regions (420 … 490 nm) and to some extent in the green (490 … 550 nm). Therefore, carotenoids extend the light spectrum for photosynthesis activity, providing absorption of 10 to 20% of solar energy photons. About 50% of the energy is absorbed in the short area—the area of high energy. These pigments are functioning as light absorbers, transferring energy of its electron in excited state to chlorophyll *a*. Reduction of carotenoid content is a negative phenomenon and indicates significant breach of all the photosynthetic apparatus.

Carotenoid content had decreased in almost 4 times (from 0.340 to 0.086 mg g^−1^) in *N. guadelupensis*, 3.4 times—in *E. canadensis* (from 0.243 to 0.072 mg g^−1^), 2.4 times—in *V. spiralis* (from 0.293 to 0.122 mg g^−1^), and 2.2 times—in *R. fluitans* (from 0.231 to 0.104 mg g^−1^). Among free-floating plants, carotenoid content had decreased as following: by 21%—in *L. laevigatum* (from 0.174 to 0.139 mg g^−1^), by 12%—in *P. stratiotes* (from 0.150 to 0.133 mg g^−1^), and by 13%—in *S. natans* (from 0.104 to 0.090 mg g^−1^).

## Conclusions

Studied aquatic plants, both submerged and free-floating, have a high capacity for removal of metal nanoparticles from aqueous solution (30–100%). Different types of plants are capable to extract only specific elements. *P. stratiotes* and *S. natans* were shown to be more preferable for phytoremediation according to their ability to uptake all studied metal nanoparticles from water.

Metal nanoparticles in aqueous medium had significantly affected the content of photosynthetic pigments, and therefore, productivity and ability of submerged hydrophytes to maintain vital functions primarily depend on a close contact of plants with nanoparticles, unlike pleuston, where only root system falls under the direct influence.

Among the investigated submerged hydrophytes, the pigment system of *N. guadelupensis* had suffered the most under the influence of nanoparticles, and *R. fluitans*—the least. Among free-floating plants, it was not possible to allocate more or less resistant species to metal nanoparticles, since their reaction was within the physiological norm and observed deviation of indicators do not cause significant changes in the vitality of plants.

Studied free-floating hydrophytes *(P. stratiotes, L. laevigatum, S. natans)* were more resistant to metal nanoparticles action in terms of stability of the photosynthetic system thus possessing higher potential for phytoremediation of contaminated water basins polluted by metal nanoparticles.

Among the studied free-floating hydrophytes, *Pistia* and *Salvinia* deserve on the special attention in terms of removal of nanoparticles. Therefore, these species are recommended for use in phytotechnological water treatment facilities.
